# YOLOv5s-FP: A Novel Method for In-Field Pear Detection Using a Transformer Encoder and Multi-Scale Collaboration Perception

**DOI:** 10.3390/s23010030

**Published:** 2022-12-20

**Authors:** Yipu Li, Yuan Rao, Xiu Jin, Zhaohui Jiang, Yuwei Wang, Tan Wang, Fengyi Wang, Qing Luo, Lu Liu

**Affiliations:** 1 College of Information and Computer Science, Anhui Agricultural University, Hefei 230036, China; 2 Key Laboratory of Agricultural Sensors, Ministry of Agriculture and Rural Affairs, Hefei 230036, China; 3 Anhui Provincial Key Laboratory of Smart Agricultural Technology and Equipment, Hefei 230036, China

**Keywords:** object detection, agricultural application, transformer encoder, multi-scale feature, collaboration perception

## Abstract

Precise pear detection and recognition is an essential step toward modernizing orchard management. However, due to the ubiquitous occlusion in orchards and various locations of image acquisition, the pears in the acquired images may be quite small and occluded, causing high false detection and object loss rate. In this paper, a multi-scale collaborative perception network YOLOv5s-FP (Fusion and Perception) was proposed for pear detection, which coupled local and global features. Specifically, a pear dataset with a high proportion of small and occluded pears was proposed, comprising 3680 images acquired with cameras mounted on a ground tripod and a UAV platform. The cross-stage partial (CSP) module was optimized to extract global features through a transformer encoder, which was then fused with local features by an attentional feature fusion mechanism. Subsequently, a modified path aggregation network oriented to collaboration perception of multi-scale features was proposed by incorporating a transformer encoder, the optimized CSP, and new skip connections. The quantitative results of utilizing the YOLOv5s-FP for pear detection were compared with other typical object detection networks of the YOLO series, recording the highest average precision of 96.12% with less detection time and computational cost. In qualitative experiments, the proposed network achieved superior visual performance with stronger robustness to the changes in occlusion and illumination conditions, particularly providing the ability to detect pears with different sizes in highly dense, overlapping environments and non-normal illumination areas. Therefore, the proposed YOLOv5s-FP network was practicable for detecting in-field pears in a real-time and accurate way, which could be an advantageous component of the technology for monitoring pear growth status and implementing automated harvesting in unmanned orchards.

## 1. Introduction

Pears are widely cultivated throughout the world [1], and China is the largest pear producer, contributing to approximately 90% of worldwide pear production. However, in traditional labor-intensive agriculture, it is often necessary to invest a large number of human resources for production activities, with a high repetition of labor tasks and low efficiency, which is not conducive to the long-term development of the pear industry. Fortunately, the rapid development of artificial intelligence technology and the innovation of general agricultural equipment has promoted the process of intelligent agriculture and provided a feasible plan for reducing human workload and promoting the transition to technology-intensive agriculture [2,3]. At present, precision agriculture technologies have become integral in collecting information without human intervention on fruit growth and health evaluation [4,5,6]. In the pear detection task, due to the similarity of color and pattern, complex background, overlapping of densely distributed target pears, ever-changing illumination conditions, and other factors, it is always greatly difficult and challenging to effectively and accurately detect target pears. It is of great significance not only to promote the development of pear growth monitoring, but also to improve the harvest efficiency and economic benefit of pears if the automatic detection of pears could be effectively implemented.

In this paper, we implemented the accurate detection of in-field pears by means of an improved YOLOv5s utilizing a transformer encoder and collaboration perception of multi-scale features. More specifically, the present work built a pear dataset, including 3680 images captured from cameras mounted on a ground tripod and a UAV platform. Specific information on the models and parameters of the charge-coupled device (CCD) camera and the unmanned aerial vehicle (UAV) platform are described in detail in Section 3.1.1. The optimized YOLOv5s-FP was then introduced with the idea of utilizing a transformer encoder to couple local and global features between feature maps and channels, and further conducting collaboration perception of multi-scale features that referred to local and global attention towards multi-scale features with different receptive fields. The contribution of this paper can be summarized into the following three aspects.

- The original cross-stage-partial module was optimized to extract non-local features through a transformer encoder, which was then fused with local features by an attentional feature fusion mechanism, achieving the mutual embedding of local features and global features.
- A modified path aggregation network oriented to collaboration perception of multi-scale features was proposed by incorporating a transformer encoder and the optimized CSP module into the paths, and new skip connections were made to transfer information between paths to enhance information exchange and prevent network degradation [7].
- Quantitative and qualitative controlled experiments were carried out to compare the detection results of pears at a variety of sizes, illuminations, and viewpoints. The experimental results indicated the beneficial impacts of the improved network on pear detection tasks in natural environments, demonstrating the good potential in assisting pear growth monitoring and automatic harvesting.

The rest of the paper is structured as follows. Section 2 introduces the construction of the dataset and describes the improved YOLOv5s network, as well as the optimization ideas of CSP and multi-scale feature collaboration perception. Section 3 presents experimental findings for the improved network and the original network for controlled detection of tasks refined at various sizes, illuminations, and viewpoints. Finally, conclusions are drawn in Section 4.

## 2. Related Works

With the deepening of the combination of artificial intelligence technology and agriculture, deep learning technology has promoted innovation in technology and pattern of many applications in agriculture [8,9]. In the field of agricultural object detection, there have been several studies geared toward fruit detection utilizing convolutional neural networks (CNNs) [10,11]. CNN-based detection networks have demonstrated that accuracy improvement in object detection could be usually performed in two classes of networks: one-stage networks and two-stage networks. Specifically, typical two-stage detectors, e.g., faster R-CNN and mask R-CNN, have been widely used in agricultural scenes, e.g., the detection and segmentation of crops and fruits [12,13]. However, there were computational time constraints associated with meeting the requirements for embedded real-time detection in practical applications. As a result, a one-stage object detection network was preferred because of its better balance among computational cost and detection accuracies [14], such as SSD [15], FCOS [16], and the YOLO series [17,18,19]. Among them, the You Only Look Once (YOLO) series were the most widely used one-stage object detection network nowadays because of its superior transferability and ease of deployment on portable devices. In this case, numerous scholars attempted to further improve the practical performance by modifying the original architecture of the YOLO networks to ensure accuracy and real-time characteristics. X. Li et al. proposed a recognition method for green peppers by combining an attention module with an adaptive spatial feature pyramid [20]. Lu et al. added the convolutional block attention module to the generic YOLOv4, improving the detection accuracy of mature apples by focusing on the target canopies [21]. Fu et al. developed one kiwifruit detection network by adding two convolutional kernels of 3 × 3 and 1 × 1 to the fifth and sixth convolution layers of the YOLOv3-Tiny [22].

Despite the positive results obtained in precedent research on fruit detection tasks, there are still deficiencies in pear detection in the natural environment; in particular, the inability to accurately detect small and occluded pears still exists. Due to the spatial resolution limitations of universal two-dimensional data acquisition equipment, it is difficult to collect spatial depth information, resulting in a large number of acquired images containing these difficult-to-detect pears. Although three-dimensional data acquisition equipment can offer options for retrieving depth information, their high economic costs and specialized knowledge requirements preclude them from meeting the requirements of practical application [23,24]. On the other hand, it might be more practical and appropriate to obtain good detection performance by improving the network structure. In general, due to low arithmetic power and data requirements, CNN-based detection frames are the preferred benchmark for object detection. However, it tends to lose feature information of small objects because of the over-convolution and pooling of CNNs for low-dimensional feature maps [25]. Additionally, as a consequence of its limited long-range perception, the features extracted from the severely occluded fruits are relatively isolated and unconnected. The feature of the same fruit might be regarded as two distinct sets of aggregation features, which might result in the misclassification of the detector and turn to the degradation of accuracy. In summary, it is critical to develop novel approaches for resolving these issues.

With a strong ability to extract contextual information, transformers have made significant advancements in the field of natural language processing in recent years [26]. Because of the exceptional contribution of transformers to long-range feature perception, some researchers have attempted to incorporate them into the field of computer vision (CV) to overcome the limitations of CNN-based networks. Dosovitskiy et al. proposed the Vision Transformer (ViT), which, for the first time, applied a transformer to a CV without modification, performing the state-of-the-art (SOTA) on large-scale datasets, but poorly when fitting small-scale datasets [27]. In comparison to the ViT, the Pyramid Vision Transformer (PVT) [28] reduced the computational complexity of large-scale feature maps by gradually shrinking the pyramid structure, while densely dividing the image pixels with high detection accuracy for dense scenes. However, because the ViT and PVT completely omitted the convolutional structure, they usually converged slowly and required large-scale datasets to fit. Thus, some combinations of transformers and CNNs have been proposed for reducing the computational cost and number of parameters [29]. D’Ascoli et al. introduced a positional self-attention mechanism equipped with convolutional inductive bias, adjusting the attention to positional and contextual information through learnable gating parameters [30]. BotNet [31] embedded multi-head self-attention (MHSA) in three bottlenecks of C5 in ResNet-50 [32] and outstood in object detection and instance segmentation tasks [33]. In summary, the architectures of the Vision Transformer contributed to the improvement of detection performance, especially for objects will a small size and serious occlusion [34]. If they were transferred to pear detection tasks, they might exhibit even greater potential in application scenarios, particularly for small and occluded pears.

A summary of the strengths and weaknesses of the aforementioned network architecture is shown in Table 1. It can be seen that the network based on the combination of CNNs and the ViT can take into account both detection accuracy and computational cost, which is beneficial to the implementation of object detection tasks in agricultural scenes with limited computing resources. Therefore, this paper explores an appropriate combination strategy and constructs an effective network for in-field pear detection.

## 3. Materials and Methods

### 3.1. Datasets

#### 3.1.1. Data Acquisition and Analysis

**Figure 1:**
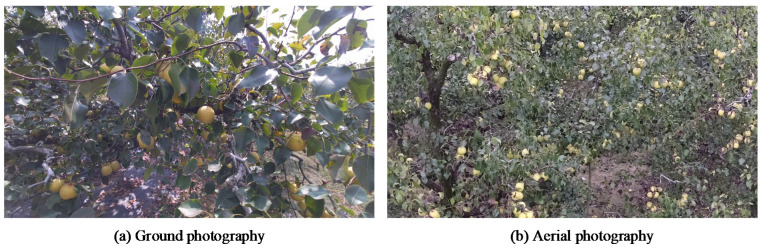
Pears in orchards from the ground and aerial viewpoints. (**a**) Image captured by the camera mounted on the ground tripod, (**b**) Image captured by the camera mounted on the UAV platform.

The data collection was conducted at a standardized pear planting demonstration base in Dangshan County, Suzhou City, Anhui Province, China, at the coordinates of 33°37′51″ N and 116°53′42″ E. The orchard is depicted in Figure 1 from a preview of the ground and aerial viewpoints. The pear trees at the base followed a standardized planting pattern with approximately equal distances between rows, providing favorable environmental conditions for data acquisition. The collection took place in September 2021, during which time the pears transitioned from the growing stage to the ripening stage. Hence, the pears were of dynamically varying sizes and colors. Data collection was carried out on sunny and cloudy days between 7:00–8:00 A.M., 10:00–11:00 A.M., 2:00–3:00 P.M., and 6:00–7:00 P.M., thus covering four different illumination conditions of weak light, normal light, and strong light in the daytime, as well as artificial light in the nighttime.

**Figure 2:**
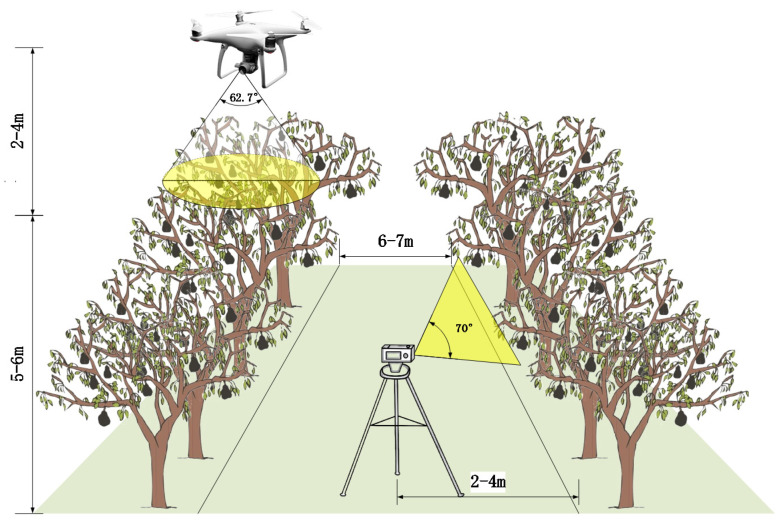
Schematic illustration of joint data acquisition.

In this paper, the images of pears were recorded by utilizing CCD cameras mounted on a ground tripod and a UAV platform. In order to minimize the demand for equipment in practical applications, in this paper, we only collect RGB images as the original data, abandoning the collection of information, such as depth, NIR, and so on. The process of joint data acquisition is illustrated in Figure 2. The CCD camera on the ground tripod was a Kodak AZ651 (Eastman Kodak Company, Rochester, NY, USA), with an effective pixel count of 20.68 million and an aperture range of F2.9–F6.7. The resolution of the image was set to 1280 × 720 pixels during the acquisition process to save memory size and to optimize the shooting frame rate. When conducting the ground photography, the CCD camera was tripod mounted and moved smoothly in parallel towards the tree trunks. The elevation angle of the camera varied ranging from 20° to 80°, whereas the distance of the camera from the tree varied from 2 m to 4 m for simulating the movement path of automatic picking platforms.

During the aerial photography, the DJI Phantom 4 (DJI technology, Shenzhen, China), was chosen as the UAV platform, with a maximum horizontal flight speed of 20 m/s, and a vertical hovering accuracy of ±0.1 m in environments with a light intensity greater than 15 Lux. The CCD camera planted on the UAV platform provided an effective pixel count of 12.4 million, a controlled rotation ranging from −90° to +30°, and a field of view (FOV) of 62.7°. The resolution was set to 1280 × 720 pixels, the same as the CCD camera on the ground. The UAV platform was deployed to fly at a height of 0–4 m above pear trees with the lens vertically downward, and the average flying speed was set to roughly 1 m/s to avoid motion blur during the photograph. In total, 7541 images were captured from ground and aerial viewpoints, containing pears of various sizes and illumination conditions. Due to the nature of the acquisition process, the speed of the acquisition device could not be adequately controlled, which might result in the emergence of images with an excessive degree of resemblance. Hence, the ORB algorithm [35] was used to select 3680 images by means of clearing and excluding those with an excessive resemblance. Ultimately, these images comprised a diverse pear dataset.

#### 3.1.2. Image Annotation and Data Enhancement

LabelImg (Windows version with Python 3.6, version 1.5.0, Heartex, San Francisco, CA, USA) was used to manually label the pears in these 3680 images, and the pear outlines were ensured to be tangent to the bounding boxes. The classification was binary, meaning that the network just had to distinguish the target pears from the background. The data was recorded in the YOLO format, with the category and the coordinates of the bounding box for each pear in each image saved in a separate TXT file. Each record in the file corresponded to a bounding box on the image and contained standardized annotation, including coordinates of the upper-left and lower-right corners. Finally, the 3680 images in the pear dataset were randomly divided into three sub-datasets: 2944 images were used for training (80%), 368 images were for validation (10%), and 368 images were for a test (10%). The training set and validation set were used to train the network and visually assess the convergence in real-time, while the test set was used to evaluate the actual performance of the pear detection network and verify the generalization ability.

Lots of pears in the field disorderly grew in crisscross branches and leaves, resulting in an extremely complex background and pear distribution. When labeling the data for training and validation, it was essential to cover pears regions of varying sizes, densities, overlapping environments, and ever-changing illuminations, which would be helpful to train the detection network with high robustness and facilitate subsequent pear detection using the trained network.

There have been a variety of data augmentation approaches that have been developed for enriching the dataset. The approaches used in this paper included random left–right flip, random up–down flip, hue saturation value (HSV) domain transformation, random blur, and mosaic enhancement [18]. These approaches were randomly selected during the image reading stage of the training process, simulating complex data distribution and noise in the environment of a natural orchard and significantly increasing the diversity and reliability of training data.

### 3.2. The Proposed YOLOv5s Improvement

#### 3.2.1. Network Architecture

The YOLOv5 was one of the most widely used object detection networks in the YOLO series. The overall structure of the YOLOv5 could be divided into three parts: backbone, neck, and head. The backbone took charge of extracting high-dimensional features, the neck took charge of extracting multi-scale low-dimensional features, and the head generated prediction boxes based on the extracted features. The YOLOv5s (small), YOLOv5m (middle), YOLOv5l (large), and YOLOv5x (extra-large) were the four sub-versions of the YOLOv5. These sub-versions were designed based on the same network structure as the main version, but with different network depths and widths. In previous experiments, it has been proved that the deeper and wider the network, the higher the detection accuracy might be [36]. However, with the increase of the depth and width of the network, the computational cost increased as well, making it unsuitable for deployment in practical application scenarios with limited computing resources. Thus, the YOLOv5s was chosen as the benchmark network in this paper.

**Figure 3:**
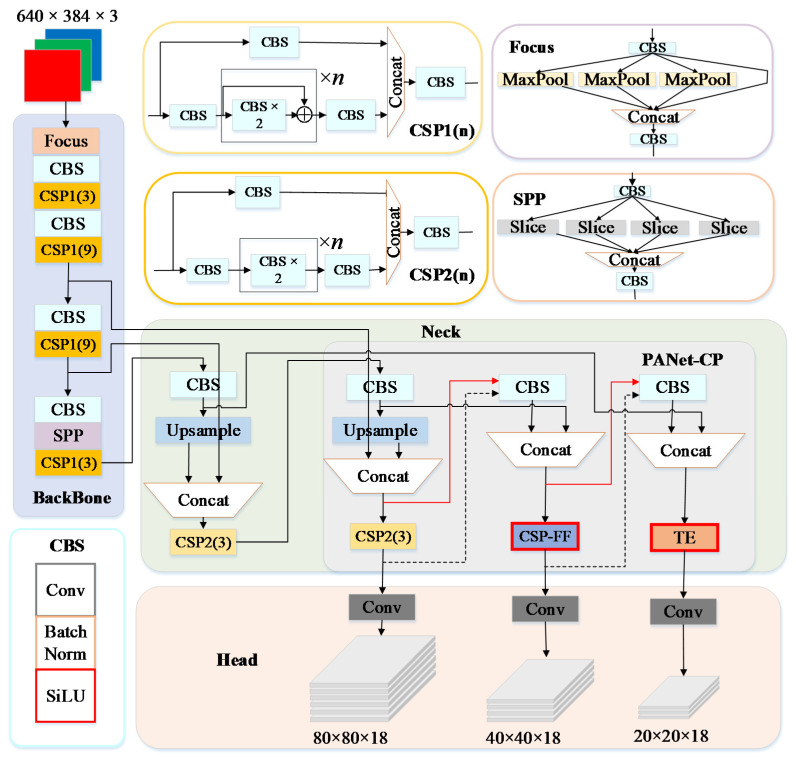
Structure of the YOLOv5s-FP (where TE corresponds to the transformer encoder, CSP-FF corresponds to feature fusion-based CSP in, and PANet-CP corresponds to collaboration perception-oriented PANet), with structural improvements marked in red boxes, new skip connections marked with red lines, and removed connections marked with dashed lines.

The YOLOv5s used CSPDarknet as the backbone and the path aggregation network (PANet) as the neck. As illustrated in Figure 3, the YOLOv5s also aggregated many essential modules, including Focus, CBS (convolution, batch normalization, and Sigmoid-ReLu), CSP (cross-stage partial module), and SPP (spatial pyramid pooling). The CSP module was designed based on CSPNet to enhance the learning ability of the network by implementing more abundant gradient combinations. It was available in two types within the YOLOv5s: CSP1 and CSP2. Each type decomposed the input feature map into a feature extraction path and a residual connection path and then fused the cross-level features. The distinction was that in the feature extraction path, CSP1 additionally used *n* residual units compared with CSP2. CSP1 was used in the backbone for extracting rich high-dimensional feature maps, whereas CSP2 was used in the neck for preventing low-dimensional feature redundancy.

To further enhance the local and global perception of multi-scale features, we improved the structure design, which could be divided into two parts: optimizing a feature fusion-based CSP structure and proposing a collaboration perception-oriented PANet. The network’s perception of local and global features was improved by establishing short-range and long-range feature dependencies and aggregating collaboration perception effectively, leading to increased robustness against background noise. Therefore, the improved YOLOv5s was designated as the YOLOv5s-FP (Fusion and Perception) and, with the application of the aforementioned improvements, it significantly improved the detection accuracy of in-field pears, particularly in the case of small pears and occluded pears.

CioU (Complete Intersection over Union) loss [37] regards the aspect ratio of the bounding box as a penalty to generate more realistic prediction bounding boxes, but when the loss converges to a linear proportion between the width and height of the prediction bounding box and the ground-truth bounding box, the width will not increase or decrease with the height. To minimize this impact, we replaced CioU loss with EioU (Efficient Intersection over Union) loss [38] as the border loss function in the head of the YOLOv5s. By clearly measuring the difference in overlap area, center point, and edge length, *EIoU* loss tackled the dilemma of existing losses and obtained a faster convergence speed and superior regression results. The formulas were as follows:

(1) $$\mathrm{IoU}=\frac{A\cup B}{A\cap B}\;$$

(2) $${\mathrm{EIOU}}_{\mathrm{Loss}}=1-\mathrm{IoU}+\frac{\rho^{2}{(b,b}^{\mathrm{gt}})}{c^{2}}+\frac{\rho^{2}{(w,w}^{\mathrm{gt}})}{C_{w}^{2}}+\frac{\rho^{\alpha }{(h,h}^{\mathrm{gt}})}{C_{h}^{2}}\;$$

where *A* and *B* represented the area of the prediction bounding box and the ground-truth bounding box, b, ${\;b}^{\mathrm{gt}}$ represented the center point of the prediction box and the ground-truth box, $\rho^{2}\left(\cdot \right)$ represented the Euclidean distance, w and h represented the width and height of the bounding box, gt represented the ground-truth bounding box, c represented the diagonal distance of the smallest outside rectangle formed by the prediction bounding box and the ground-truth bounding box, and $C_{w}$ and $C_{h}$ represented the width and height of the smallest enclosing bounding box covering the prediction bounding box and ground-truth bounding box.

#### 3.2.2. Feature Fusion-Based CSP

It has been discovered that the convolution operation had deficiencies in perceiving and modeling global features, which made it difficult to constitute long-range dependencies in the spatial domain and channel domain. Nowadays, deep neural networks became deeper and wider through the stacking of convolution, but dense or huge convolution kernels might filter out features that were vital for small targets, resulting in a reduction in detection accuracy. In addition, the receptive field of small convolution kernels was relatively local, whereas the features of severely occluded objects were relatively discrete. Therefore, it was more likely to misidentify them as two objects when using small convolution kernels.

To solve these problems, we introduced the transformer encoder. As we know, the transformer encoder was first put forward to deal with natural language processing, where it was used to encode long-range semantic information in one-dimensional sequences, allowing for the establishment of long-range dependencies. The transformer encoder has been proven to apply to the field of computer vision as well; namely, it could handle two-dimensional strongly correlated data. Encoding a two-dimensional matrix in a two-dimensional spatial domain could be accomplished by partitioning the image into numerous patches and downscaling each patch to a one-dimensional sequence. The use of the transformer encoder, with the ability to construct global features, made it possible to extract long-range features while preserving the spatial structure of the data. A reasonable combination of the features extracted from convolution and the transformer encoder would improve local and global perception simultaneously.

**Figure 4:**
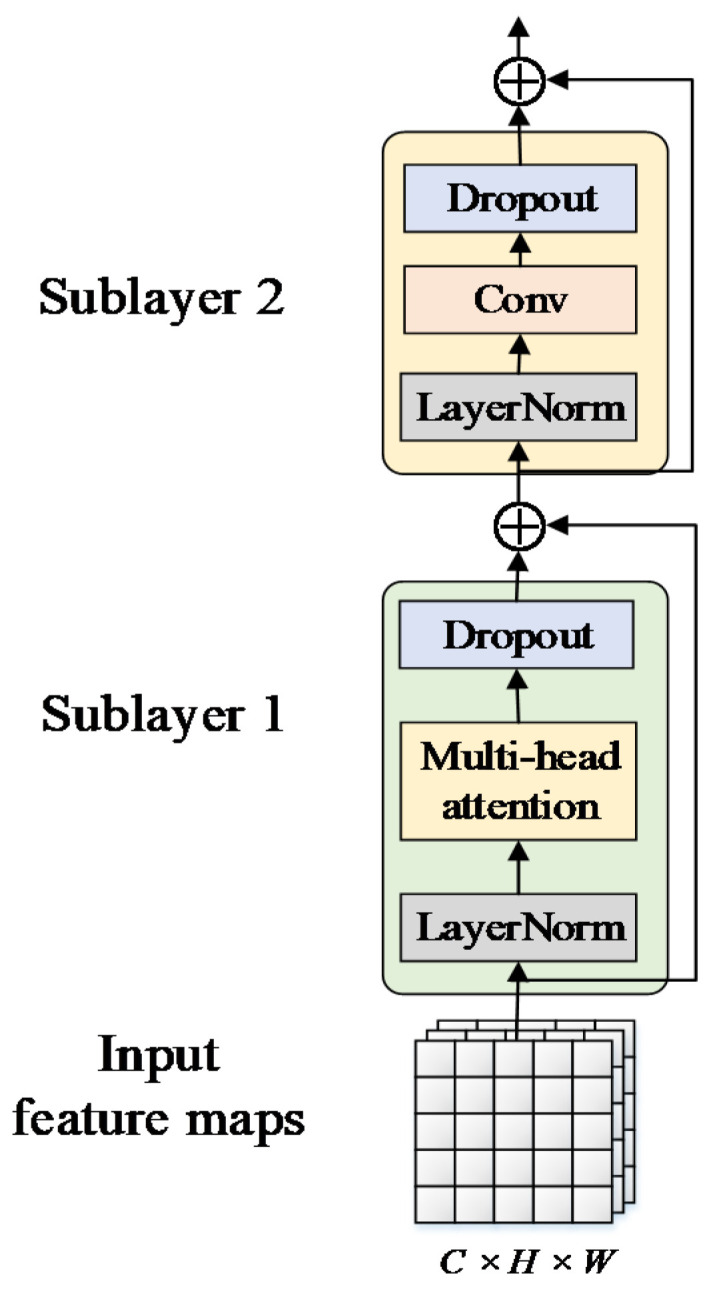
Structure of the improved transformer encoder (TE).

In Visformer (a vision-friendly transformer) [39], one improved transformer encoder was proposed by replacing multi-layer perceptron layers designed in the ViT with convolutional layers, making it friendlier for visual recognition tasks and showing a promising performance when it was evaluated on small datasets. Drawing on the above work, we made similar improvements to the transformer encoder to make it more effective for visual detection tasks. As illustrated in Figure 4, the improved transformer encoder was composed of two sublayers: the first was a multi-head attention sublayer, while the second was a feed-forward layer. The multi-head attention was similar to multi-kernel convolution in CNNs, which extracted information from multiple dimensions by dividing the parameter matrix into multiple subspaces. The feed-forward layer remapped the subspaces to the target space by convolution, achieving information aggregation of multi-head attention, while the dropout layer was used to disregard some of the concealed nodes to prevent overfitting and increase generalization ability. The output feature map contained long-range dependencies of global features, which could be embedded into any part of the network due to invariant scale. In comparison to convolution, the transformer encoder enlarged the receptive field of the feature map at the expense of slightly increased computational cost, hence delivering more rich semantic information for downstream detection tasks.

The transformer encoder was more adept at extracting long-range features, reflecting complex spatial transformers and long-range dependencies. However, it ignored local feature details, which decreased the discriminability between background and foreground. The convolutional operation was more concerned with integrating local information, but experienced difficulty in capturing global features. We referred to the two styles of features with deviations in the distribution of perceptual domains extracted from the transformer encoder and convolution as global and local features. The receptive fields of these two-type features were highly complementary, and they were also inextricably correlated, which meant sufficient aggregation of these features would hold the potential to consistently yield feature maps of higher quality. Traditionally, the typical method for fusing the two-style features was to concatenate the feature maps directly. However, the direct concatenation of these features might allocate the features with fixed weights regardless of the variance of contents, resulting in the scale inconsistency issue among input features and a decrease in the quality of output feature maps.

**Figure 5:**
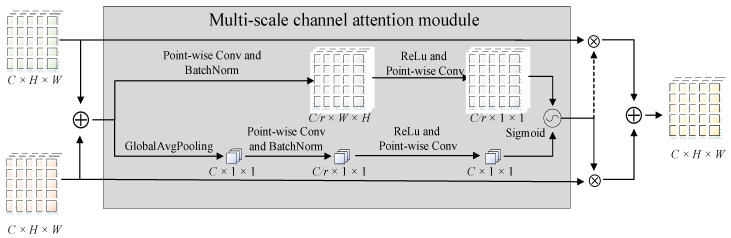
Structure of attentional feature fusion, where the dashed line denotes 1−M(X⊕Y).

As a result, we introduced the attentional feature fusion mechanism (AFF) [40], which could add local channel contexts to the global channel-wise statics. The structure of AFF was depicted in Figure 5. With the combination of a multi-scale channel attention module and long-skip connections, it could aggregate local and global contexts and achieve the fusion of feature maps with larger and smaller receptive fields. In this study, the given two-style feature maps are as follows: $X,Y\in {\mathbb{R}}^{C\times H\times W}$, where X referred to the CNN-extracted feature maps and *Y* referred to the transformer encoder-extracted feature maps. The equations for AFF were defined as follows:

(3) Output=M(X ⊕ Y) ⊗ X+(1 − M (X ⊕ Y)) ⊗ Y 

where ⊕ denoted broadcasting addition, ⊗ denoted the element-wise multiplication, M denoted multi-scale channel attention mechanism, and $M\left(X\right)\in {\mathbb{R}}^{C\times H\times W}$ denoted attentional weights generated by M.

**Figure 6:**
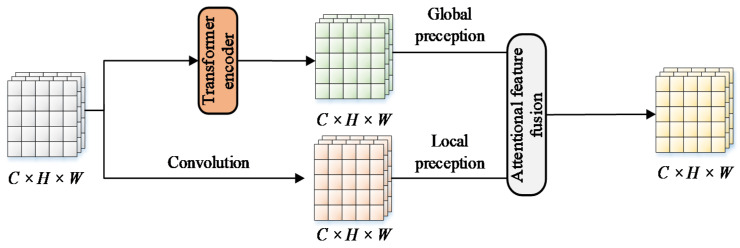
Structure of feature fusion-based CSP (CSP-FF).

In the structure of the YOLOv5s, CSP2 in the PANet decomposed the input feature map into two routes through the use of 1 × 1 convolution kernels. One of the routes was connected for residuals, while the other was in charge of implementing feature extraction. The feature maps delivered to the feature extraction route were extracted by *n* bottlenecks, which might result in certain undesirable effects such as overfitting, gradient disappearance, and accuracy degradation. Aiming to exploit the advantages of two-style features and overcome the limitations in the receptive field, we designed the feature fusion-based CSP (CSP-FF). As a solution, we replaced the bottlenecks with the transformer encoder in the original CSP2 and implemented convolution and encoding in parallel, and the structure of CSP-FF is shown in Figure 6. In this case, the two pathways output the feature map with a smaller receptive field and the feature map with a larger receptive field, which corresponded to the X and Y of AFF. Following the integration and redistribution of the fused information, the overall distribution of the output feature maps tended to be uniform after transmission to AFF, offering more valuable aggregated features in both local and global receptive fields. The final output was adjusted using another 1 × 1 convolution kernel to balance the channels while maintaining a consistent scale between the input and output. CSP-FF was utilized to replace CSP2 inside the PANet to alleviate the problems arising from complex background noise and scale variation of in-field pears.

#### 3.2.3. Collaboration Perception-Oriented PANet

In the YOLOv5s, PANet was employed as the neck network to provide large, medium, and small-scale feature maps with the head for implementing object detection. After downsampling, PANet resampled the feature maps and obtained the information before and after sampling by horizontal skip connections. High-dimensional semantic information was transferred to low-dimensional semantic information, which improved the representation of low-dimensional information. Meanwhile, low-dimensional semantic information was transferred to high-dimensional semantic information, which improved the utilization of the underlying information. However, the information loss caused by the convolution of low-dimensional features was amplified because upsampling exacerbated the information loss caused by the convolution of high-dimensional features. This resulted in a reduction in detection accuracy for small and occluded pears. In order to make full use of the feature information of each dimension, it was necessary to modify some modules and connections of PANet to minimize information loss caused by network degradation. Therefore, if one expected to enhance the overall perception of the network, it was also of great importance to formulate specific perception approaches for each scale of the feature maps to enhance the overall perception of the network.

**Figure 7:**
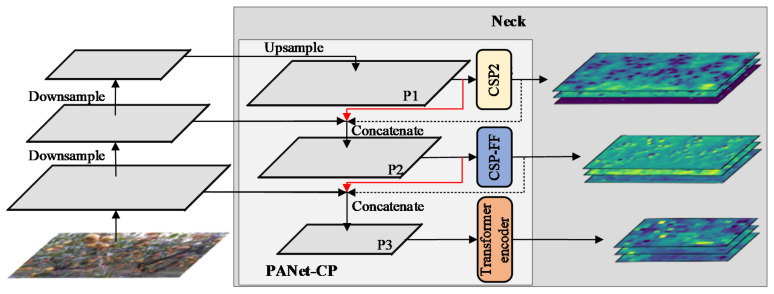
Structure of collaboration perception-oriented PANet (PANet-CP), with new skip connections marked with red lines and removed connections marked with dashed lines.

PANet could be classified as P1, P2, and P3 based on the size of the extracted feature maps, which corresponded to high-resolution, medium-resolution, and low-resolution feature maps, respectively. These feature maps with different resolutions provided a diverse set of multi-scale feature information, which was beneficial to the extraction of feature descriptors of different dimensions. In this paper, we embedded a transformer encoder and CSP-FF into PANet to improve the ability of local and global feature extraction, forming the collaboration perception with different receptive fields towards multi-scale features. The structure of the collaboration perception-oriented PANet (PANet-CP) is illustrated in Figure 7.

The transformer encoder was embedded into P3 to replace the original CSP2 for processing low-resolution feature maps and achieving low-dimensional information refinement. On the other hand, the accuracy of the network was more dependent on the quality of the medium-resolution feature maps, which should contain more spatial information. Therefore, by substituting CSP-FF for the original CSP in P2, it was possible to acquire and integrate global and local information concurrently. P1 retained its structure in order to reduce the loss of local information caused by the frequent use of the transformer encoder to extract features, as well as to avoid the explosion of network parameters. The proposed feature perception approach was called multi-scale feature collaboration perception, allowing the network to accomplish detection for different objects at different scales and providing a solution to precisely embed local and global features into each other.

In addition, the methods of information propagation were improved for preventing network degradation. In the original PANet, the feature map received by P2 was extracted by multiple CSP2s in P1, and the feature map received by P3 was extracted by multiple CSP2s in P1 and P2, forming a cascade relationship that caused the network to become rather dense. Additionally, due to the incorporation of CSP-FF and the transformer encoder, the computational complexity of the neck network was increased. Here, to lessen the network depth and achieve valid information transmission, two new skip connections were made for bypassing the feature extraction module in P1 and P2. The feature maps passed to P2 and P3 bypassed one and two feature extraction modules, respectively, which reduced the risk of losing some important features for medium and small objects, as well as avoided the decrease in information transmission efficiency caused by bypassing too many modules. By the modification of information propagation, the output multi-scale feature maps of PANet-CP contained denser local and global information, offering a good ability to implement real-time and efficient detection tasks of pears in the orchard.

## 4. Experimental Results and Analysis

### 4.1. Network Training

The training platform included one NVIDIA GTX 2080Ti GPU (NVIDIA Corporation, Santa Clara, CA, USA) with 12GB of memory and a six-core Intel Xeon E5-1650 processor (3.60 GHz) (Intel, Santa Clara, CA, USA). On the training platform, the system environment was comprised of CUDA (version 10.2, NVIDIA Corporation, Santa Clara, CA, USA), CUDNN (version 7.6.5, NVIDIA Corporation, Santa Clara, CA, USA), and Python (version 3.8, Python Software Foundation, Wilmington, DE, USA), with the deep learning framework Pytorch (version 1.8.0, Meta, Menlo Park, CA, USA). With the network trained on the training set of pears using eight images as a batch, the training loss was updated per iteration for a total of 200 epochs. To economize on the time and data required to train a network from scratch to achieve the desired level of operational accuracy, we employed transfer learning by retraining on the pre-trained weights from the MS COCO dataset. The network was optimized using SGD, the initial learning rates of all layers were set to 0.01, the weight decay rate was set to 0.00048, and the momentum factor was initially set to 0.937 and decayed to 1E-4. The images were scaled to a resolution of 640 × 640 pixels and input which accelerated the network training, in which the size of small pears was further reduced, making accurate detection more challenging.

### 4.2. Network Evaluation

Comprehensive performance evaluation metrics were required for object detection networks in order to evaluate the network and thus provided feedback for manual optimization of the training hyperparameters. The precision, recall, *F*1-*score*, and average precision (AP) were used as evaluation metrics for the YOLOv5s-FP in this paper. The *F*1-*score* was composed of two components: precision and recall. Precision was the ratio of correctly predicted positives to all positive predictions, recall was the ratio of correctly predicted positives to all actual positive predictions, and *F*1-*score* was the harmonic mean of precision and recall. Equations (4)–(6) give the calculation formulas of precision, recall, and *F*1-*score*. *TP* (True Positive) denoted the number of predicted positive samples, *FP* (False Positive) denoted the number of predicted positive but negative samples, and *FN* (False Negative) denoted the number of predicted negative but positive samples.

(4) $$\mathrm{Precision}=\frac{\mathrm{TP}}{\mathrm{TP}+\mathrm{FP}}$$

(5) $$\mathrm{Recall}=\frac{\mathrm{TP}}{\mathrm{TP}+\mathrm{FN}}$$

(6) $$F1-\mathrm{score}=\frac{2*\mathrm{Precision}*\mathrm{Recall}}{\mathrm{Precision}+\mathrm{Recall}}$$

The calculation formula for AP was shown in Equations (7) and (8). Here, *r* denoted the integration variable for calculating the integral product of precision and recall. ${\mathrm{AP}}_{50}$ was defined as the average value of precision when the intersection over union (*IoU*) was 0.50 and ${\mathrm{AP}}_{50:95}$ was the average value of all ten *IoU* thresholds with a uniform step size of 0.05 in the range of [0.50, 0.95].

(7) $$\mathrm{AP}=\overset{1}{\underset{0}{\int }}(\mathrm{precision}*\mathrm{recall})\mathrm{dr}$$

(8) $${\mathrm{AP}}_{50:95}=\frac{1}{10}{(\mathrm{AP}}_{50}{+\mathrm{AP}}_{55}+\cdots {+\mathrm{AP}}_{90}{+\mathrm{AP}}_{95})$$

The trained YOLOv5s-FP was validated and tested to verify the performance of the network. Images were scaled to the same resolution as the training input. The performance of the network was represented by the values of the five evaluation metrics defined in Equations (4)–(8) through validation, which provided quantitative evidence for early intuitive evaluation of the improvement effects. The results of the test reflected the final performance of the network, and the numerical findings were considered the controlled object for the controlled experiments.

### 4.3. Quantitative Performance of the YOLOv5s-FP

This paper aimed to develop a pear detection network with higher accuracy and less computational cost. Hence, the YOLOv5s-FP should be accurate and fast enough to detect a variety of pears in the natural environment, particularly in the case of small and occluded pears. Accordingly, the performance of the network should be discussed in three straightforward aspects: accuracy, detection time, and computational cost. Apart from the mentioned accuracy evaluation metrics $AP_{50}$, $AP_{50:95}$, and F1-score, we also measured the detection time, memory usage, and FLOPs of the network. Table 2 summarizes the quantitative performance of 368 images in the test set on these indicators. It could be concluded that the YOLOv5s-FP achieved accurate pear detection with less detection time and computational cost. The memory usage and FLOPs of the YOLOv5s-FP were 50.01 MB and 18.2 G, respectively, which was suitable for use on portable devices with limited computing power and storage space.

### 4.4. Comparison with the YOLO Series Networks

Table 3 presents the quantitative evaluation results of the YOLOv5s-FP, as well as the other five typical YOLO networks. The small sub-versions of these networks were selected for making comparisons in order to reduce the interference of different sub-versions on the results, ensuring that the experimental results were of reference significance. The comprehensive evaluation metrics were identical to those described previously in terms of accuracy, detection time, and computational cost. It could be observed that the $AP_{50}$, $AP_{50:95}$, and *F*1-*score* of the YOLOv5s-FP attained the highest level, outperforming other typical networks on all three metrics of accuracy, particularly the YOLOv3s, YOLOv4s, and YOLOv5s. In comparison to the YOLOXs, YOLOv5s-FP achieved a certain improvement in accuracy with less detection time and computational cost. This was reflected in the reduction of 14.22 MB and 8.6 G in memory usage and FLOPs, respectively. The experimental results demonstrated that by using the transformer encoder and collaboration perception of multi-scale features, the proposed network could improve detection accuracy without significantly increasing the computational cost. It is worth mentioning that the YOLOv5s-FP achieved better performance compared to the other evaluated networks, except that in terms of detection time, the YOLOv5s-FP was slightly (2 ms) slower than the original YOLOv5s, but it did not suppress the real-time performance, even in application scenarios with extremely limited computing power.

### 4.5. Ablation Experiments

**Figure 8:**
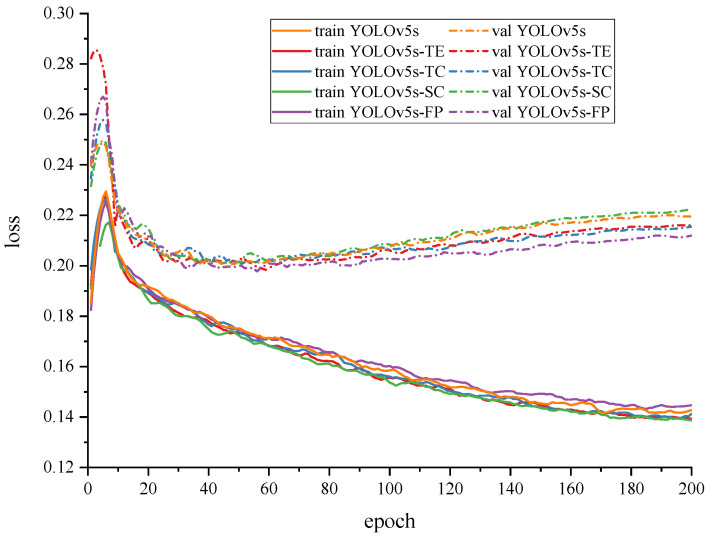
Comparison of coordinate loss of ablation experiments on the training and validation set.

Although the effectiveness of the YOLOv5s-FP based on the transformer encoder and collaboration perception-oriented PANet has been demonstrated above, the underlying mechanisms of the improvements remained unknown. Hence, ablation experiments were carried out to ascertain the effectiveness of these mechanisms and their influence on pear detection. Specifically, four ablation networks, named the YOLOv5s, YOLOv5s-SC, YOLOv5s-TE, and YOLOv5s-TC, were established for controlled experiments. Based on the YOLOv5s and YOLOv5s-SC-modified PANet with new skip connections, the YOLOv5s-TE replaced CSP2 in P3 with a separate transformer encoder and the YOLOv5s-TC replaced CSP in P2 with a separate CSP-FF. The loss of the YOLOv5s-FP and four ablation networks on the training and validation sets are illustrated in Figure 8. The figure shows that the loss of both training and validation sets for all networks exhibited a single peak, i.e., the loss value appeared to increase during the early training period and then resumed its decreasing trend as the epoch progressed. The analysis of the loss of training set revealed that the loss value of the YOLOv5s-SC increased to the least degree and reached the minimum at the end of the training, whereas the loss value of the YOLOv5s-FP was higher than other networks. The validation set loss was then analyzed, and while the peak of the YOLOv5s-SC remained the lowest, the validation set loss at the end of the training was also the greatest compared to the other four ablation networks. The validation set loss of the YOLOv5s-FP was significantly lower than that of other ablation networks, and the curve was smoother. Therefore, it could be concluded that the new skip connections realized the reduction in network depth and effective information transmission, preventing network degradation to a certain extent and making the network converge faster. However, the YOLOv5s-SC performed well on the training set but poorly on the validation set, revealing that its generalization ability was weakened and overfitting might occur. Both the transformer encoder and CSP-FF offered the ability of the YOLOv5s-FP to prevent overfitting and the loss of the training set and validation set was relatively stable and low. However, the transformer encoder caused a significant increase in validation loss at the early stage, implying that the network was more difficult to fit. The validation loss of the YOLOv5s-FP was the lowest, indicating that it had the strongest generalization ability and was more suitable to handle unevenly distributed data acquired from natural environments.

From the experiment result for each network in Table 4, it can be concluded that new skip connections resulted in a greater increase in accuracy at a relatively less cost in terms of detection time and computational cost. The transformer encoder and CSP-FF provided greater gains in accuracy but at the expense of increased detection time and computational cost. The transformer encoder had the primary effect of increasing the memory usage of networks, whereas the CSP-FF had the primary effect of increasing detection time consumption. The YOLOv5s-FP combined three improvements into the PANet to form new components, named PANet-CP. It can be seen that the YOLOv5s-FP is significantly superior to other ablation networks in all three accuracy metrics: $AP_{50}$, $AP_{50:95}$, and *F*1-*score*. Although the YOLOv5s-FP increased detection time and computational cost, the boosted value remained small and less than that of most typical object detection networks in Table 3. In summary, the abovementioned improvements contributed to better performance and stronger stability of the YOLOv5s-FP in terms of detection accuracy, detection time, and computational cost.

### 4.6. Visual Performance Comparison

In the above quantitative experiments, the YOLOv5s-FP has been demonstrated to be superior to the original YOLOv5s and other typical networks of the YOLO series. However, the qualitative performance in practical application scenarios remain to be further explored. In natural environments, the growth of pears was rather disordered. To maximize harvesting efficiency and monitoring ability during the ripening period, it was typically expected that the harvesting could be conducted throughout the day. Therefore, the detectors applied in agricultural management were required to have a strong ability to adapt to the changes in environmental factors and to implement fruit detection during the day and night. We made comparisons of visual detection performance between the original YOLOv5s and YOLOv5s-FP in three aspects: different pear sizes, illuminations, and viewpoints, to ascertain the practical utility of the networks. In the following part, representative images were selected from the test set as a comparison, with qualitative detection results for the original YOLOv5s and YOLOv5s-FP being illustrated. The confidence threshold was set at 0.25, which meant that bounding boxes with a confidence level of less than 0.25 were filtered out. Meanwhile, the *IoU* threshold was set at 0.5, which meant those bounding boxes with lower repetition to the ground truth bounding box were filtered out.

#### 4.6.1. Comparison of Test Results at Different Pear Sizes

Various locations of image acquisition resulted in a variation in the size of the pears in the photographs. Usually, the pears that were closest to the camera were easier to be detected, as they were relatively larger within the acquired images and thus enjoyed a higher picking priority. Pears farther away from the camera appeared in relatively small size within the obtained images, and the accurate detection of these pears provided additional information for making decisions, e.g., the planning of subsequent picking paths. Spatial information about the dense regions of unpicked pears would be recorded and arranged for the upcoming collecting strategy with the assistance of three-dimensional perception equipment. To further refine the dataset and to provide more detailed data support for the paper, the pears were classified into three categories according to the size of their bounding boxes. Specifically, those pears with a radius of fewer than 10 pixels were regarded as small pears, those with a radius of more than 10 pixels but less than 25 pixels were regarded as medium pears, those with a radius of more than 25 pixels were regarded as large pears, and those with a radius of fewer than 5 pixels were omitted. There were a total of 166,580 annotated pears, including 41,596 small pears, 96,950 medium pears, and 28,034 large pears. It was worth noting that the refinement of the pear dataset was performed only when conducting the comparison experiment of test results at different sizes, and the aforementioned categorization patterns were not employed during the training.

**Figure 9:**
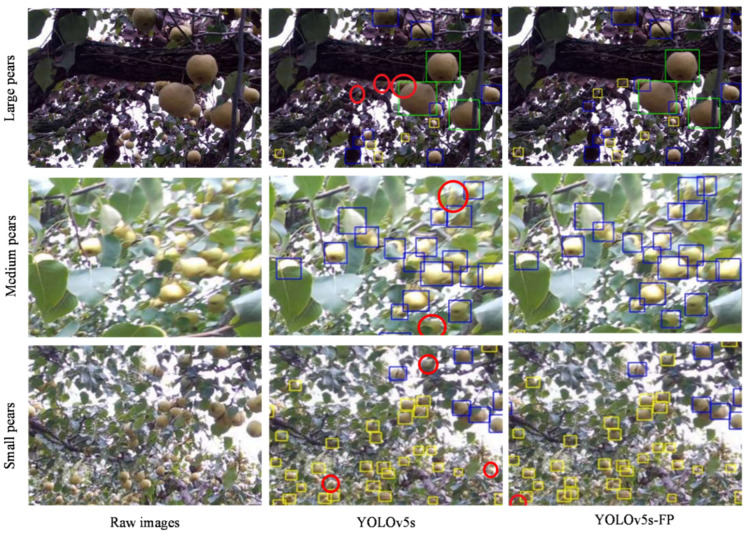
Comparison of pear detection results at different pear sizes, where missing pears were marked in red circles, and pears inside green, blue, and yellow boxes were referred to as large, medium, and small pears, respectively.

Figure 9 depicts the detection results for the original YOLOv5s and YOLOv5s-FP on representative example images, containing dense regions of large, medium, and small pears. As illustrated in this figure, both the original YOLOv5s and YOLOv5s-FP detected large and naked pears well. However, the YOLOv5s-FP was better at focusing on small pears and those pears occluded by leaves, branches, and each other in the detection scenario, demonstrating high adaptability to size changes. Table 5 presents the detection results of the quantitative experiments in the form of numerical values, with ${\mathrm{AP}}_{50}$, ${\mathrm{AP}}_{50:95}$, and *F*1-*score* as the evaluation metrics. It could be seen that the detection accuracy of the YOLOv5s-FP for small-size pears had greatly increased as compared to the original YOLOv5s, increasing by 5.1% for ${\mathrm{AP}}_{50}$, 3.8% for ${\mathrm{AP}}_{50:95}$, and 5.3% for *F*1-*score*. Additionally, a numerical improvement was also observed for medium and large pears, with ${\mathrm{AP}}_{50}$ increasing by 1.6% and 1.1%, respectively. This showed that overall detection accuracy has improved, as the YOLOv5s-FP could correctly differentiate pears in the background. The table and images provided strong support for the conclusion that the YOLOv5s-FP was superior to the original YOLOv5s in detecting pears at different sizes, benefiting from the joint utilization of local and global features and collaboration perception of multi-scale features that took full advantage of the two-style feature maps.

#### 4.6.2. Comparison of Test Results under Different Illumination Conditions

In natural environments, the ever-changing illumination conditions could easily lead to variation in data distribution, resulting in the degraded performance of object detection networks, which might not be conducive to the long-term development of smart robots. The adaptability of the networks to illumination changes was regarded as an important aspect of the network performance evaluation. The test set contained 368 images captured in a variety of illumination conditions, including weak illumination, normal illumination, strong illumination in the daytime, and artificial illumination in the nighttime. A handheld LED rod of 400 lumens was placed 2–3 m from the image acquisition target to make artificial illumination. Here, non-normal illumination was considered, including weak, strong, and artificial illumination. They accounted for 60% of the total duration in the dataset which covered complex scenes and background noise in the natural environment.

**Figure 10:**
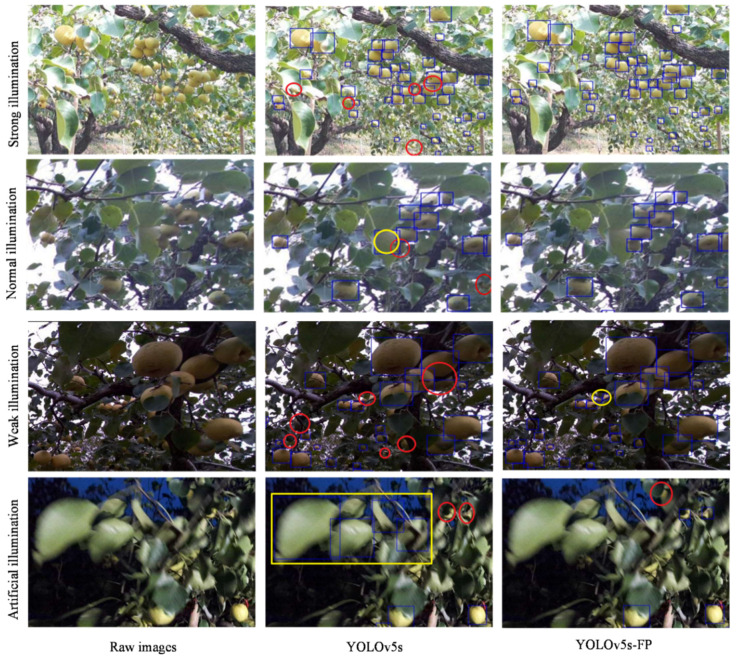
Comparison of pear detection results under different illumination conditions, where missing pears were marked in red and false detection pears were marked in yellow.

Figure 10 shows the qualitative detection results of the original YOLOv5s and YOLOv5s-FP under the aforementioned illumination conditions in the test set, and Table 6 summarizes the quantitative detection results in terms of $AP_{50}$, $AP_{50:95}$, and *F*1-*score*. Under strong and weak illumination, the YOLOv5s-FP showed significantly higher detection accuracy with more small pears in severe occlusion. The reason was the fact that the YOLOv5s-FP achieved the collaboration perception of multi-scale features, and the local feature and global feature were constituted, maintaining superior detection performance even in the presence of varying background noise. Similar results were obtained under normal and artificial illumination, while the false positive rate of the YOLOv5s-FP was lower compared to the original YOLOv5s. It was apparent that under artificial illumination with insufficient lighting, it could easily occur that the human vision system mistook leaves for pears. Similarly, the original YOLOv5s had the characteristics of misidentifying leaves as pears. In contrast, the YOLOv5s-FP had a stronger ability to capture information about the spatial structure of objects and, thus, it could effectively avoid misidentification. In comparison to the original YOLOv5s, the $AP_{50}$, $AP_{50:95}$, and *F*1-*score* of the YOLOv5s-FP increased under artificial illumination by 5.4%, 3.7%, and 6.7%, respectively, which further assured the advanced robustness and resistance of the YOLOv5s-FP to changes in illumination. In summary, by combining the transformer encoder and multi-scale feature collaboration perception, the YOLOv5s-FP was able to focus on the structural features of pears, which were less susceptible to color and posture changes and more robust to the dynamic background.

#### 4.6.3. Comparison of Test Results in Different Viewpoints of UAV

In view of the development of UAV technology, it has shown to be a good prospect in various industries [41]. As we intended to verify its feasibility in agriculture and the adaptability of the network to the change of viewpoints, further controlled experiments were conducted on images captured by the UAV in the side and bird’s-eye viewpoints. The horizontal viewpoint obtained from the side of trees was considered to be the side viewpoint, while the UAV image taken from the vertical ground above trees is considered a bird’s-eye viewpoint. Usually, the greater camera distances away from trees contributed to a smaller overall proportion of pears in the images, and the disordered growth of branches and leaves significantly hindered detection ability and caused high false detection.

**Figure 11:**
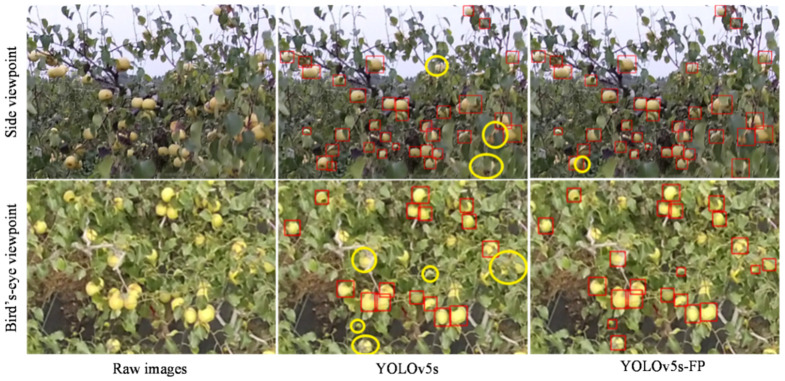
Comparison of pear detection results in different viewpoints of UAV, where missing pears were marked in yellow circles.

In this section, the qualitative detection results of drone images captured from two viewpoints were compared between the original YOLOv5s and YOLOv5s-FP, as illustrated in Figure 11. The quantitative detection accuracy in terms of $AP_{50}$, $AP_{50:95}$, and *F*1-*score* are presented in Table 7. The first row of Figure 11 showed the scene from the side viewpoint, with plenty of small and occluded pears hidden in leaves and branches. As can be seen, the YOLOv5s-FP achieved better detection results, with fewer missing pears and a lower false-positive rate. The YOLOv5 and YOLOv5s-FP both had good detection results for naked pears, but the YOLOv5s-FP showed a stronger ability to detect small and occluded pears. It was further supported by the data in the first row of the table, which increased by 3.4%, 2.0%, and 1.6% for $AP_{50}$, $AP_{50:95}$, and *F*1-*score* compared to the side viewpoint. Similar experimental results were obtained from the bird’s eye viewpoint, where some extremely small and occluded pears were missed by the original YOLOv5s but successfully detected by the YOLOv5s-FP, with $AP_{50}$, $AP_{50:95}$, and *F*1-*score* increments of 2.8%, 1.5% and 1.8%, respectively. Although the false detection rate of the two networks both increased, which might be caused by the complexity of the background, the YOLOv5s-FP was relatively better at completing the detection task and was more suitable in practical detection scenarios. In conclusion, it could be drawn that the YOLOv5s-FP would have a better potential for detecting small and occluded pears in complex orchard scenarios, as well as being extended to more agricultural management activities, such as pear growth status monitoring and orchard yield estimation.

## 5. Conclusions

It is still challenging for traditional object detection networks to effectively detect in-filed pears with disordered distribution and complex background noise, particularly for those small and occluded ones in natural orchards. For the purpose of efficiently detecting pears, a novel pear detection network named the YOLOv5s-FP (Fusion and Perception) was proposed by improving the neck of the original YOLOv5s in combination with a transformer encoder, attentional feature fusion mechanisms, and new skip connections. By means of achieving mutual embedding of local and global features and collaboration perception of multi-scale features, the proposed network achieved a higher accuracy on pear detection tasks than most networks of the YOLO series with fewer requirements of detection time and computation resources. Additionally, qualitative comparison experiments visually showed that the YOLOv5s-FP improved the overall perception results of pears with differences in size, illumination condition, and viewpoint in practical application scenarios, demonstrating that it could function as an integral part of monitoring pear growth and implementing automated harvesting. The proposed YOLOv5s-FP provided a feasible idea for the innovation of fruit detection technology in modern orchards. Further work includes the collection of continuous pear images at different growth and phenological stages for validating its performance and further improving its robustness, which might be more helpful for conducting pear growth status monitoring and automated harvesting activities.

## Figures and Tables

**Table 1:** The comparison of the strengths and weaknesses of the different network architectures.

Network Architecture	Strengths	Weaknesses
Based on CNNs	Low computational cost, strong real-time performance, and easy to deploy.	The detection accuracy of small and occluded objects can be improved.
Based on the ViT	It is beneficial to the detection of small and occluded pears, and the accuracy can be significantly improved.	The computational cost and memory usage are too high, which is not conducive to real-time detection.
Based on a combination of CNNs and the ViT	Taking into account both accuracy and computational cost, it is helpful in the detection of small and occluded pears.	An inappropriate combination can still result in a significant increase in computational cost and memory usage.

**Table 2:** Overview of accuracy, detection time, and computational cost of the YOLOv5s-FP.

Networks	$AP_{50}$ (%)	$AP_{50:95}$ (%)	*F*1-*Score* (%)	Detection Time (ms)	Memory Usage (MB)	FLOPs (G)
YOLOv5s-FP	96.12	68.22	89.73	13.2	50.01	18.2

**Table 3:** Accuracy, detection time, and computational cost of the YOLOv5s-FP and typical networks of the YOLO series.

Networks	$AP_{50}$ (%)	$AP_{50:95}$ (%)	*F*1-*Score* (%)	Detection Time (ms)	Memory Usage (MB)	FLOPs (G)
YOLOv3s	86.51	59.42	83.72	16.1	120.32	157.1
YOLOv4s	89.98	62.83	85.62	20.2	246.34	137.2
YOLOv5s	89.09	63.09	84.35	11.2	13.70	16.4
YOLOXs	92.62	64.31	86.23	14.3	64.23	26.8
YOLOv5s-FP	96.12	68.22	89.73	13.2	50.01	18.2

**Table 4:** Ablation experiments of the YOLOv5s-FP.

Networks	$AP_{50}$ (%)	$AP_{50:95}$ (%)	*F*1-*Score* (%)	Detection Time (ms)	Memory Usage (MB)	FLOPs (G)
YOLOv5s	89.09	63.09	84.35	11.2	13.7	16.3
YOLOv5s-SC	89.56	63.39	84.48	11.2	13.9	16.8
YOLOv5s-TE	92.92	63.07	84.61	11.7	42.2	17.8
YOLOv5s-TC	94.58	64.33	86.45	12.4	21.2	17.9
YOLOv5s-FP	96.12	68.22	89.73	13.2	50.0	18.2

**Table 5:** Detection accuracy of two networks at different sizes.

Networks	Small			Medium			Large		
	$AP_{50}\;(\%)$	$AP_{50:95}\;(\%)$	*F*1-*Score* (%)	$AP_{50}\;(\%)$	$AP_{50:95}\;(\%)$	*F*1-*Score* (%)	$AP_{50}\;(\%)$	$AP_{50:95}\;(\%)$	*F*1-*Score* (%)
YOLOv5	82.8	51.3	84.2	95.1	65.2	87.1	96.2	88.6	89.2
YOLOv5s-FP	87.9	55.1	89.5	97.5	69.3	90.2	97.3	89.5	92.1

**Table 6:** Detection accuracy of two networks under different illumination conditions.

**Illumination Conditions**	YOLOv5s-FP			YOLOv5s			Count of Images	Count of Annotated Pears
	$AP_{50}\;(\%)$	$AP_{50:95}\;(\%)$	*F*1-*Score* (%)	$AP_{50}\;(\%)$	$AP_{50:95}\;(\%)$	*F*1-*Score* (%)		
Strong illumination	95.8	66.2	89.3	90.2	62.3	84.2	80	2066
Normal illumination	97.5	73.2	93.1	95.3	71.9	91.9	124	3690
Weak illumination	96.4	69.9	92.2	92.2	66.2	89.5	96	2630
Artificial illumination	90.8	65.1	88.8	85.4	61.4	82.1	68	784

**Table 7:** Detection accuracy of two networks in different viewpoints of the UAV.

**Viewpoint** **of UAV**	YOLOv5s-FP			YOLOv5s			Count of Images	Count of Annotated Pears
	$AP_{50}\;(\%)$	$AP_{50:95}\;(\%)$	*F*1-*Score* (%)	$AP_{50}\;(\%)$	$AP_{50:95}\;(\%)$	*F*1-*Score* (%)		
Side viewpoint	96.8	71.4	92.8	93.4	69.4	91.2	1392	262,624
Bird’s-eye viewpoint	83.3	61.0	82.1	80.5	59.5	80.3	648	185,722

## Data Availability

Not applicable.
